# Genome-Wide Identification of Small RNAs in the Opportunistic Pathogen *Enterococcus faecalis* V583

**DOI:** 10.1371/journal.pone.0023948

**Published:** 2011-09-02

**Authors:** Kouki Shioya, Charlotte Michaux, Carsten Kuenne, Torsten Hain, Nicolas Verneuil, Aurélie Budin-Verneuil, Thomas Hartsch, Axel Hartke, Jean-Christophe Giard

**Affiliations:** 1 Laboratoire de Microbiologie de l'Environnement, EA956-USC INRA 2017-IFR146 ICORE, University of Caen, Caen, France; 2 Institute of Medical Microbiology, Justus-Liebig-University, Giessen, Germany; 3 Genedata AG, Basel, Switzerland; East Carolina University School of Medicine, United States of America

## Abstract

Small RNA molecules (sRNAs) are key mediators of virulence and stress inducible gene expressions in some pathogens. In this work we identify sRNAs in the Gram positive opportunistic pathogen *Enterococcus faecalis*. We characterized 11 sRNAs by tiling microarray analysis, 5′ and 3′ RACE-PCR, and Northern blot analysis. Six sRNAs were specifically expressed at exponential phase, two sRNAs were observed at stationary phase, and three were detected during both phases. Searches of putative functions revealed that three of them (EFA0080_EFA0081 and EFB0062_EFB0063 on pTF1 and pTF2 plasmids, respectively, and EF0408_EF04092 located on the chromosome) are similar to antisense RNA involved in plasmid addiction modules. Moreover, EF1097_EF1098 shares strong homologies with tmRNA (bi-functional RNA acting as both a tRNA and an mRNA) and EF2205_EF2206 appears homologous to 4.5S RNA member of the Signal Recognition Particle (SRP) ribonucleoprotein complex. In addition, proteomic analysis of the ΔEF3314_EF3315 sRNA mutant suggests that it may be involved in the turnover of some abundant proteins. The expression patterns of these transcripts were evaluated by tiling array hybridizations performed with samples from cells grown under eleven different conditions some of which may be encountered during infection. Finally, distribution of these sRNAs among genome sequences of 54 *E. faecalis* strains was assessed. This is the first experimental genome-wide identification of sRNAs in *E. faecalis* and provides impetus to the understanding of gene regulation in this important human pathogen.

## Introduction

Some RNA molecules such as riboswitches, transfer-messenger RNA (tmRNA) and small non-cording RNAs (sRNAs) act usually as post-transcriptional regulators in bacteria [1]. sRNAs have become increasingly recognized as an emerging class of gene expression regulators for cellular processes, stress response and virulence genes and their transcription is tightly regulated and induced by distinct environmental conditions [2]. Bacterial sRNAs found on chromosomes are typically 50–400 nucleotides in length and frequently encoded in intergenic regions (IGRs). They may bind to the imperfect complementary sequence of the ribosome binding region of the target mRNA, which is often encoded at separate loci, thus inhibiting 30S ribosomal subunit association and translational initiation [1], [3]. In some Gram positive and Gram negative species such as *Escherichia coli* [4] and *Listeria monocytogenes* [5], the formation of sRNA-mRNA duplex requires the RNA chaperon protein Hfq [6], [7] leading to an increase of mRNA degradation by ribonucleases such as RNase E and RNase III [2]. Some sRNAs located in plasmids and phages act as antisense RNAs on *cis*-encoded mRNAs and mainly control replication initiation, conjugation efficiency and transposition [8], [9]. In addition, plasmid-encoded sRNAs, called *hak/sok* system of *E. coli* plasmid R1 [10] and *par* system of *Enterococcus faecalis* pAD1 [11], stabilize their host plasmids by programming for death any cell that loses the plasmid [9], [12].

In recent years, several bioinformatic approaches have been performed to identify putative sRNAs in bacterial genomes including *E. coli*, *L. monocytogenes*, *Bacillus subtilis* and *Pseudomonas aeruginosa*, and identified more than 200 sRNAs [13]. Recently, Livny *et al.* predicted *in silico* over 45,000 sRNA candidates from 932 bacterial genomes [14]. In parallel, different experimental strategies including cDNA sequencing, shotgun cloning and isolation from RNA-protein complex have been performed and sometimes lead to the discovery of new transcripts [15], [16]. Tiling microarrays are powerful approaches to identify sRNAs on a genome-wide scale. Thus large numbers of sRNA candidates have been found in *Caulobacter crescentus*, *Streptococcus pyogenes*, *S. pneumoniae*, and *L. monocytogenes* genomes [17], [18], [19], [20].

*E. faecalis* is a human commensal Gram-positive bacteria as well as one of the leading causes of hospital acquired infections in United States and Europe [21]. The first whole genome sequence of *E. faecalis* V583 strain (the first vancomycin resistant enterococci identified in U.S.A.) was determined in 2003 and 53 more sequences are now publically available [22]. *In silico* study performed by Livny *et al.* led to the prediction and annotation of 17 putative sRNA-encoding loci in *E. faecalis* [14]. Surprisingly, in comparison with *E. coli* and *B. subtilis*, the number of predicted sRNAs in V583 is roughly 10-fold lower, suggesting that this number is likely under-estimated. Recently, 45 sRNAs and 10 putative mRNAs have been identified in *E. faecalis* using *in silico* prediction combined with “5′tag-RACE” [23].

In this work, we developed custom-made tiling microarrays containing only IGRs of *E. faecalis* V583 chromosome and plasmids, and first performed hybridization with RNA extracted from exponential and stationary-phase cells. Fifty-three statistically significant positive signals were detected and the 12 putative sRNAs most highly expressed were selected for further characterization. Transcription of these candidates under several stress conditions was then analyzed.

## Materials and Methods

### Bacterial strain and growth conditions

All experiments were performed with *E. faecalis* V583 strain [24]. For our first tiling array assays, cells were grown at 37°C in M17 0.5% glucose medium and collected at exponential phase (OD_600_ = 0.5) and at 24 h stationary phase. Growth in BHI medium with or without aeration was tested. Cells were collected at exponential phase (OD_600_ = 0.5), onset of starvation (OD_600_ = 2) and late stationary phase (24 h). For experiments under stress conditions, bacterial cells were grown to OD_600_ = 0.3 in M17 medium and H_2_O_2_ (2 mM), lactic acid (pH 5.5), or bile salts (BS) (0.08%), were added before an additional 30 min incubation at 37°C. For the growth in urine and serum, *E. faecalis* was inoculated into human urine or horse serum (Eurobio, Courtaboeuf, Fr) during overnight. Cells were then pelleted and resuspended into fresh urine or serum for 3 hours at 37°C. Urine collected from four healthy volunteers was pooled, centrifuged and sterilized by filtration (0.22 µm-pore sizes). Written consent from all participants involved in our study was obtained. French CPP (Comité de Protection de Personnes) exempted this study from review because volunteers were informed of the goal of this study, no health information was collected and no biological analysis was performed on these samples.

### RNA extraction and tiling microarray hybridization

Total RNA was extracted using Trizol reagent (Invitrogen, Carlsbad, CA) as described by Toledo-Arana *et al.* [20], with the following modifications. Bacterial cells were resuspended into 200 µl of “max bacterial enhancement reagent” (Invitrogen) and transferred into micro tubes containing glass beads and 400 µl acid phenol (Ambion, Austin, TX). Bacteria were mechanically lysed using Mixer Mill 200 (30/s, 30 min, Retsch, Haan, Germany). After centrifugation for 10 min at 14,000 g at 4°C, aqueous phase was transferred to 2 ml tubes containing 1 ml Trizol reagent, mixed and incubated for 5 min at room temperature (RT). 200 µl chloroform was added, mixed gently and incubated for 3 min at RT. Tubes were centrifuged for 15 min at 12,000 g at 4°C and aqueous phase was transferred into 2 ml tubes containing 200 µl chloroform, mixed gently and centrifuged again. RNAs contained in the aqueous phase were precipitated by addition of 500 µl isopropanol and incubated for 10 min at RT. After centrifugation, RNA pellets were washed with 75% ethanol and dried at RT. Purified RNA pellets were resuspended in DEPC-treated pure water.

To enhance detection sensitivity by enriching of sRNAs and removing non-sRNA, 10 µg RNA were fractionated using flashPAGE Fractionator (Applied Biosystems, Foster City, CA). Fractionated RNA was labelled using mirVana labelling kit (Applied Biosystems) and then hybridized onto the tiling array. 1745 “big intergenic regions (IGR)” (more than 49 nt) and 1070 “small IGR” (from 1 to 49 nt) have been deduced from *E. faecalis* V583 genome sequence. 50 nt long probes with an overlap of 15 nt were loaded on our IGR custom-made tiling arrays. rRNA and tRNA probes were used as positive control showing signal intensity of hybridization at least 10 fold the threshold level. Since the values of intensity observed in apparent untranslated regions were between 1000 and 2000, 2000 was used as threshold. For each experiment (one sample per growth condition) two chips were used; one corresponding to the forward, and one to the reverse strand. Production, hybridization and data collecting were carried out by Febit biomed GmbH Company (Heidelberg, Germany). The detection was carried out using streptavidin phycoerythrin at different exposure times. Data analyses and visualization were performed by Genedata Phylosopher Business Group (Basel, Switzerland). We have deposed the raw data at GEO/ArrayExpress under accession number GSE28741, we can confirm all details are MIAME compliant.

### 5′ and 3′ rapid amplification of cDNA ends (RACE) analysis

For these analysis, new RNA samples were prepared as described above. 5′ RACE was performed using 2nd Generation 5′/3′ RACE kit (Roche, Mannheim, Germany) according to the manufacturer's instructions. For polymerase chain reactions (PCR), we used Go Taq polymerase and its buffer (Promega, Madison, WI). The primers used for cDNA synthesis, and for the PCR reactions are listed in Table S1.

For 3′ RACE experiments, total RNAs were treated with poly(A) polymerase (Epicentre, Madison, WI) for 15 min at 37°C. After 3′ end RNA poly(A) tailing, cDNA was synthesized with QuantiTect Reverse Transcription kit (Qiagen, West Sussex, UK) and oligo(dT)-anchor primer supplied in 5′/3′ RACE kit. cDNA products were directly used as templates for PCR performed with the gene-specific primers (Table S1) and the respective PCR anchor primer. After sequencing, 5′ and 3′ ends sequences were determined.

### Northern blotting

Northern blots were performed according to standard procedures [25]. Five µg of total RNA were separated on 1.2% formaldehyde agarose gel and transferred to Hybond N^+^ membrane (Amersham, UK). 0.1–1 kb RNA Marker (Sigma, USA) was used to estimate the sizes of RNA bands. DNA oligonucleotides probes (Table S1) were labeled with α^32^P-ATP using Terminal Deoxnucleotidyl Transferase Recombinant enzyme (Promega) as recommended by the manufactured protocol. Membranes were prehybridized for 1 h in hybridization buffer (0.25 M NaH_2_PO_4_, 0.25 M Na_2_HPO_4_, 5% SDS) at 45°C, followed by addition of labelled probes and overnight hybridization at 45°C. Membranes were washed with washing buffer (3×SSC buffer, 0.2% SDS) for 5 min at RT and were then exposed to storage phosphor screen (Packard Instrument Company, Mariden, CT) for 3 h.

### *In silico* analysis

Rho-independent terminators were predicted with TransTerm (http://nbc11.biologie.uni-kl.de/framed/left/menu/auto/right/transterm/) [26]. Blast searches between *E. faecalis* strains were carried out using a species-level BLAST database (http://www.ncbi.nlm.nih.gov/). The Rfam database was employed to determine putative functions of sRNAs (http://rfam.sanger.ac.uk) [27]. In order to predict target genes for the identified sRNAs sRNATarget (http://ccb.bmi.ac.cn/srnatarget/) [28] and IntaRNA (http://rna.informatik.uni-freiburg.de:8080/IntaRNA.jsp) [29] servers were used.

### Construction of EF3314_EF3315 sRNA deletion mutant

For the deletion assay, a DNA fragment containing ligated upstream (869 bp) and downstream (839 bp) sequences of the EF3314_EF3315 sRNA, was cloned into plasmid pMAD [30] (see Table S1 for primers used). 1 µg of recombinant plasmid was finally used to transform competent cells. After electroporation, 300 µl of cell suspension was plated onto GM17 agar containing 50 µg ml^−1^ of erythromycin and X-Gal (100 µg ml^−1^). Plates were incubated for 48 hours at 30°C. A few dark blue colonies were obtained and analysed for presence of the plasmid by PCR using primers *madR* and *madF* (Table S1). Some blue colonies were then cultured twice in GM17 liquid medium with erythromycin (50 µg ml^−1^) at 45°C over-night. In the next step, the cultures were used to inoculate (0.05% v/v) GM17 liquid medium without antibiotic. The tubes were incubated for 6 hours at 30°C followed by incubation at 45°C over-night. This step was repeated 2 to 3 times. Serial dilutions of the culture were plated on GM17 agar containing 100 µg ml^−1^ of X-Gal and incubated for 48 hours at 45°C. White colonies were then isolated on GM17 agar with or without erythromycin. Antibiotic sensitive clones were analysed by PCR on the presence of a deleted sRNA.

### Two-dimensional protein gel electrophoresis and protein identification

Protein samples from wild type and ΔEF3314_EF3315 mutant cells harvested in exponential growth phase were performed as described by Giard *et al.* [31]. First dimensional electrophoresis was carried out using 17 cm ReadyStrip™ IPG Strips (pH 4–7) and Protean®IEF Cell apparatus (Bio-Rad Laboratories, Richmond, CA, USA) as recommended by the manufacturer. Second dimensions were performed in 14% polyacrylamide gels without stacking gel using the Millipore Investigator™ 2-D electrophoresis system (Millipore, Bedford, MA, USA) as described by Giard *et al.* [31]. 2-D gels were then stained using Coomassie Blue. Spots of interest were excised from the gel, and peptides were digested by trypsin as described by Budin-Verneuil *et al.* [32]. An electrospray ion trap spectrometer (LCQ DecaXP, ThermoFinnigan, San Jose, CA, USA) coupled on line with HPLC was used for peptides analysis. Mass spectrometry were acquired in a mode that alternated a full MS scan (mass range: 400–1600) and a collision induced dissociation tandem mass spectrometry (MS/MS) of the most abundant ion. Data were analysed using the sequest algorithm incorporated with the ThermoFinnigan BioWorks software.

## Results and Discussion

### Tiling microarray-based identification of *E. faecalis* sRNAs

Tiling microarray has become a comprehensive approach to sRNA discovery. Identification of sRNA candidates transcribed by *E. faecalis* V583 was undertaken with two samples of cells harvested in mid-log growth phase and stationary phase after 24 h of incubation at 37°C in M17 glucose media. Analysis of IGRs tiling microarray data revealed 53 regions with intensity values of hybridization five fold higher than signals from apparent untranslated regions. Importantly, only one (see below) of these putative sRNAs identified by microarray was also predicted by bioinformatic approach as performed by Livny *et al.* [14]. This low overlap between microarray and *in silico* analysis is consistent with that observed in other bacteria [18]. These data show that computational and experimental methods are two complementary ways to identify sRNAs. As carried out for identification of sRNAs from *S. pneumoniae* using tiling arrays, we choose a stringent intensity cutoff to avoid false positives for identifying short length RNA [19]. Using a threshold of intensity of ten fold the background level led to the identification of 12 putative sRNAs (Table 1). No experimental evidence (neither sequence from RACE-PCR nor signal on Northern blot) was obtained for one of them (EF0940_EF0941). Since the IGR between EF0940 and EF0941 is only 51 bp in length, the corresponding probe putatively hybridized with the transcription product of EF0941. Thus, the candidate has been excluded from our study. The 11 other candidates that hybridized in specific intergenic regions were selected for further detailed characterization.

**Table 1:** sRNAs in *E. faecalis* V583 detected by tiling microarray.

	Intergenic Region	Left gene	sncRNA strand	Right gene			Size	Flanking genes		Expression value^a^		Expression ration(Expo/Stat)
					start	stop	(nt)			Expo	Stat	
sRNAs expressed at exponential phase												
A.	EF3314_EF3315	←	←	←	3201675	3201582^b^	94	EF3314:cell wall surface anchor family protein		65025.9	1249.6	52
						3201535^b^	141	EF3315:triphosphoribosyl-dephospho-CoA synthase				
B.	EF0820_EF0822	←	←	→	784383	784014	370	EF0820:rplY; 50S ribosomal protein L25/general stress protein Ctc		37086.5	1376.8	26.9
								EF0822:HAD (haloacid dehalogenase) superfamily hydrolase				
C.	EFA0080_EFA0081	→	←	→	63478^c^	63423^b^	99	EFA0080:UvrC family transcriptional regulator	RNAI	37537.9	3062.9	12.3
								EF0081:hypothetical protein				
D.	EF1368_EF1369	←	→	←	1345556	1346183	628	EF1368:hypothetical protein		35465.0	3058.9	11.6
								EF1369:Cro/Cl family transcriptional regulator				
								EF1370:drug resistance transporter, EmrB/QacA family protein				
E.	EF0408_EF0409	→	→	←	381297	381708	412	EF0408:PTS (phosphotransferase system) system, IIA component	RNAI	47418.0	11648.3	4.1
								EF0409:hypothetical protein				
F.	EF0605_EF0606	←	→	←	569151	569329	179	EF0605:hypothetical protein		41977.3	11288.0	3.7
								EF0606:Dps (DNA-binding protein from starved cells) family protein				
sRNAs expressed at stationary phase												
G.	EF1097_EF1098*	→	←	←	1067257	1066894	364	EF1097:hypothetical protein	tmRNA	3390.8	63399.5	0.05
								EF1098:hypothetical protein				
H.	EF0869_EF0871	←	←	→	829525	829052	474	EF0869:Cro/Cl family transcriptional regulator		2655.4	47286.9	0.06
								EF0871:cation transpoter E1–E2 family ATPase				
I.	EF0136_EF0137	→	←	→	137278	137066^d^	>213	EF0136:hypothetical protein		1755.7	28560.7	0.06
								EF0137:nucleotidyl transferase domain-containing protein				
sRNAs expressed at exponential and stationary phase												
J.	EFB0062_EFB0063	→	←	→	55834	55623^b^	212	EFB0062:UvrC family transcriptional regulator	RNAI	49218.4	52343.1	0.94
								EFB0063:replication control protein PrgN				
K.	EF2205_EF2206	→	←	←	2119382	2119296	87	EF2205:hypothetical protein	4.5S	41604.0	55672.1	0.75
								EF2206:cytidine/deoxycytidylate deaminase family protein				

a : Intensity of hybridization from the intergenic probe showing the highest signal in exponential or stationary phase.

b : Computer prediction of the putative 3′ end (using TransTerm software).

c : 5′ end corresponding to the 5′ end of probe.

d : 3′end corresponding to the 3′ end of probe.

### Experimental validation of 11 sRNAs in *E. faecalis*

**Figure 1:**
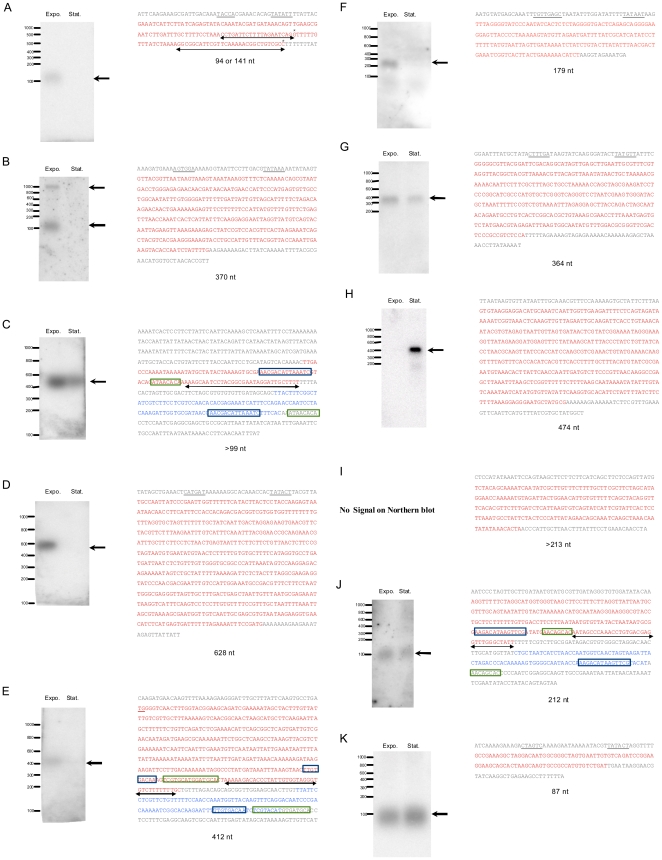
Northern blots and sequences of sRNAs (A: EF3314_EF335, B: EF0820_EF0821, C: EFA0080_EFA0081, D: EF1368_EF1369, E: EF0408_EF0409, F: EF0605_EF0606, G: EF1097_EF1098, H: EF0869_EF0870, I: EF0136_EF0137, J: EFB0062_EFB0063 and K: EF2205_EF2206). RNA was isolated from cells at exponential (Expo) and stationary (Stat) phases. Northern blot analyses were performed using α^32^P-labelled probes. Arrows on Northern blot picture indicate the sRNAs corresponding bands. The transcriptional start sites and terminators of sRNAs were determined by 5′ RACE and 3′ RACE or by *in silico* analysis using TransTerm software. The putative −10 and/or −35 promoter sequences are underlined, and the sRNA sequence is written in red letters. Putative 3′-ends of EF3314_EF335 sRNA (panel A) is indicated by stars (*). The 3′-end of the sequenceof EF0136_EF0137 (panel I) mentioned here corresponds to the 3′-end of the tiling array probe. Black arrows in the sequence indicate the predicted terminators. The *fst* gene is written in blue letters and direct repeats “a” and “b” (DRa and DRb) of *par* system are blue and green boxed, respectively (panels C, E, and J).

One of the main goals of this study was to determine the sequence and the expression pattern of the 11 selected sRNA candidates. First, using a new RNA preparation, we performed Northern blot analysis to confirm the transcription of these RNAs during exponential growth phase and stationary phase and to determine the approximate size of each candidate. We observed a transcript for 10 out of the 11 candidates tested. Six of them (EF3314_EF3315, EF0820_EF0821, EFA0080_EFA0081, EF1368_EF1369, EF0408_EF0409 and EF0605_EF0606) were specifically expressed during exponential phase (Figure 1A–F); 1 sRNA (EF0869_EF0870) was specifically expressed after 24 h of starvation (Figure 1H); and 3 (EF1097_EF1098, EFB0062_EFB0063 and EF2205_EF2206) were detected in comparable amounts in both phases (Figure 1G, J, K). These expression patterns were in good agreement with the results of tiling microarray except for EF1097_EF1098 which was much more expressed in stationary phase than under growing conditions on our chips. For unexplained reasons, no signal has been detected for EF0136_EF0137 (Figure 1I) by Northern blot analysis under our experimental conditions.

In order to determine the exact sequence of each sRNA candidate we identified the transcriptional start sites by 5′-RACE except for EFA0080_EFA0081 for which no result was gained. The 3′ ends of the transcripts were obtained either by 3′-RACE (Figure 1B, D, E, F, G, H, K, Table 1) or by combining transcript length data deduced from the Northern blots and computational prediction of transcriptional terminators [26] (Figure 1A, C, J, Table 1). Since neither putative terminator nor experimental data of the 3′ end of EF0136_EF0137 (Figure 1I) were obtained, the end of the sequence mentioned corresponds to the 3′ end of the tiling array probe. 5′-3′ RACE data of EF0820_EF0821 did not correlate to Northern blot results. From RACE-PCR, a 370 nt long sRNA was deduced that is larger than the predicted size (app. 100 nt) from Northern blot (using probe hybridizing on the 5′ region), suggesting that the large EF0820_EF0822 transcript was processed to short sRNA by modification of its 3′ end. Except for EF0820_EF0822, where the 99 last nucleotides correspond to the beginning sequence of EF0820, we could not identify obvious coding sequences (CdS), i.e. ORFs (open reading frames) with start codons connected to putative ribosome-binding sites in reasonable distances (around 8 nucleotides) inside the other sRNA candidates. Nevertheless, definitive exclusion of the presence of CdS in these regions needs experimental verification.

Altogether, the length range of the identified sRNAs was 87–628 nucleotides and the deduced sequences and promoter regions of the 11 sRNAs are shown in Figure 1. In comparison with sRNAs identified by Fouquier d'Hérouel *et al*. [23] using *in silico* prediction and “5′tag-RACE” strategy, only four overlap with our sRNA candidates (EF0605_EF0606, EF1097_EF1098, EF0869_EF0871, and EF2205_EF2206 corresponding to *ref*25C, *ssr*A, *ref*19C, and *ffs*, respectively). This shows that several techniques as well as different growth conditions (see below) are necessary for more exhaustive identification of sRNAs.

### Features of sRNAs

As previously mentioned, an antisense RNA regulated addiction module named “*par*” system was described on the *E. faecalis* plasmid pAD1 [9]. The components of this toxin-antitoxin (TA) system are antisense RNA (RNA II) and its target, RNA I encoding the peptide toxin Fst. Such systems play a crucial role in plasmid stability by killing any daughter cells that fail to inherit a copy of the plasmid. Three putative sRNAs identified in our study (EFA0080_EFA0081 in pTEF1, EFB0062_EFB0063 in pTEF2, and EF0408_EF0409 in the chromosome) corresponded to the RNAI components of the TA systems already identified in *E. faecalis* V583 by Weaver and coworkers [11]. As shown in Figure 1 (C, E, J), RNA I (including *fst* toxin gene) and RNA II homologues had two direct repeat sequences and shared the same bidirectional terminator. One interesting question concerns the role of *par* addiction module located on the bacterial chromosome. Several studies revealed various roles such as in mobile element stability or stress response [12], [33], [34]. As pointed out, in the case of *par*_EF0409_ (including EF0408_EF0409 sRNA), its association with genes encoding phosphotransferase components homologous to a mannitol transport system suggests a potential function in nutritional uptake [11].

Northern blot and tiling microarray showed that EF1097_EF1098 was expressed in both growth and stationary phases and we were able to determine the exact sequence of this sRNA (Figure 1G). EF1097_EF1098 corresponds to *E. faecali*s tmRNA (*ssrA*) that is a unique bi-functional RNA acting as both a tRNA and an mRNA. It functions as the rescue system of ribosomes stalled on aberrant mRNAs and adds a peptide tag to nascent polypeptides for directed proteolysis (named *trans*-translation) [35], [36]. tmRNA is universally conserved and is one of the most abundant RNA in the cells [37]. It has not only an important role in mRNA turnover but also likely in monitoring protein folding (for review see [35]). Mutations that inactivate tmRNA are lethal for some species (ie, *Neisseria gonnorhoeae*, *Haemophilus influenzae*, *Shigella flexneri*) or, for others, affect bacterial physiology such as virulence (ie, *Salmonella enterica*, *Yersinia pseudotuberculosis*) or stress response (ie, *E. coli*, *B. subtilis*) [35], [37]. Determination of the impact of tmRNA deletion in *E. faecalis* is under investigation in our laboratory.

We used the Rfam database (a collection of non-coding RNA families) to determine the putative functions of characterized sRNAs [27]. We found that EF2205_EF2206 sRNA matched with the Signal Recognition Particle (SRP) functional category. SRP is a ribonucleoprotein complex that targets proteins for secretion through co-translational process and is composed of protein Ffh and 4.5S RNA in prokaryotes. Our analysis revealed that EF_1700 gene (*ffh*) product and EF2205_EF2206 correspond to the two components of the SRP in *E. faecalis*. Interestingly, a recent study demonstrated that mutation of the gene encoding 4.5S RNA in *S. pyogenes* (phylogenetically related to *E. faecalis*) results in reduction of virulence [38].

In order to predict target genes of the other sRNAs identified in this study, we performed *in silico* analysis (Table 2, Table S2). Two different softwares were used for a more precise identification. sRNATarget server is based on the Naive Bayes probabilistic method and take RNA secondary structure profile as the feature [28]. The second, IntaRNA, predicts interactions between two RNA molecules, and the scoring is based on hybridization free energy and accessibility of the interaction sites in both molecules [29]. Numerous putative target genes were obtained by combination of these two approaches (from 9 for EF3314_EF3315 to 81 for EF0136_EF0137) (Table 2, Table S2). *In silico* prediction (Table S2) as well as sequence analysis suggested antisense activity for EF1368_EF1369 and EF0136_EF0137. Indeed, EF1369 mRNA sequence, encoding a putative transcriptional regulator, was fully complementary to EF1368_EF1369 sRNA. Likewise, the first 136 nucleotides of EF0136_EF0137 were complementary with the beginning sequence of EF0137 mRNA. The combined *in silico* data constitute hypothetical regulons for the sRNA candidates that need to be experimentally verified.

**Table 2:** Number of putative target genes.

	Number of mRNA candidate		
sRNA	sRNATarget (score>0.9)^a^	IntaRNA^b^	common^c^
EF3314_EF3315	75	31^d^	9
EF0820_EF0822	176	213^d^	44
EF1368_EF1369	876	97^e^	72
EF0605_EF0606	210	85^d^	24
EF0869_EF0871	494	318^d^	62
EF0136_EF0137	1252	92^e^	81

a : http://ccb.bmi.ac.cn/sRNAtarget [28].

b : http://rna.informatik.uni-freiburg.de:8080/IntaRNA.jsp [29].

c : list of genes is in Table S2.

d : cut-off <−10 kcal/mol.

e : cut-off <−15 kcal/mol.

**Figure 2:**
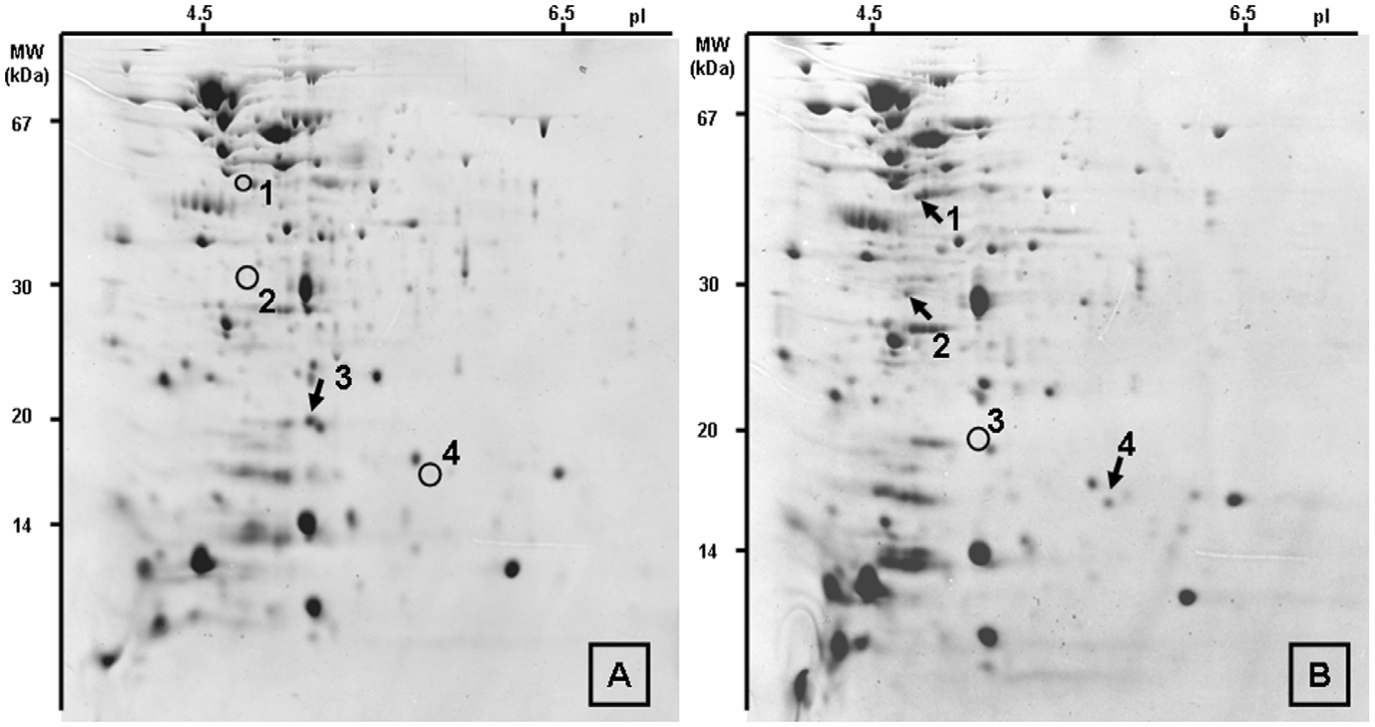
Two-dimensional gel electrophoresis of proteins from *E. faecalis* V19 (A) and ΔEF3314_EF335 mutant (B). Arrows indicate polypeptides that are detected in one gel but not in the other. The position of the polypeptides absent in a given gel are indicated by circles.

In general, sRNAs act at the post transcriptional level of regulation [1], [3]. Then, in order to observe a putative influence of one sRNA in *E. faecalis*, proteomic approach was undertaken comparing profiles of the ΔEF3314_EF3315 mutant and the parental strain. Two-dimensional gel electrophoresis of proteins from growing *E. faecalis* V19 and ΔEF3314_EF3315 mutant strains are shown in Figure 2. From two distinct experiments we observed that intensity of 4 spots were reproducibly different between the two strains. Numbers 1, 2, and 4 were only present in the mutant whereas number 3 was only seen in the wild type (Figure 2). By mass spectrometry, after extraction of proteins from the gel, we identified these polypeptides. Spots 1, 2, 3, and 4 correspond to DnaK (EF_1308, 63 kDa), ribosomal protein S1 (EF_1548, 43 kDa), ribosomal protein L6 (EF_0221, 19 kDa), and translation elongation factor Tu (EF_0221, 43 kDa), respectively. However, molecular weight (MW) deduced from the gels (around 45 kDa, 30 kDa, 15 kDa, for peptides 1, 2, and 4, respectively) did not correlated with the expected sizes. Therefore, peptides indentified from the mutant samples likely corresponded to protein degradation products. On the other hand, MW of spot number 3, which is absent in the mutant, was estimated at around 20 kDa in good accordance with the calculated size of the intact protein (19 kDa). These combined results suggested that EF3314_EF3315 might be involved in the turnover of some abundant proteins in *E. faecalis*, especially from the translational apparatus.

### Expression of sRNAs in different stress conditions

Generally, the expression of sRNAs are tightly regulated and induced by specific environmental condition [2]. We then performed tiling arrays with new RNA samples in order to analyze the transcription of sRNAs previously characterized under 11 different conditions of growth some of which may correspond to stresses encountered during intestinal colonization or during the infectious process (see Material and Methods). Expression patterns of the 11 sRNAs under H_2_O_2_, BS, and acid stress conditions, during growth in presence or absence of O_2_ and in serum and urine is presented in Table 3. EF0408_EF0409, EFA0080_EFA0081 and EFB0062_EFB0063, identified as members of TA systems were highly expressed at different stages of growth with oxygen (Table 3). Physiological significance of the induction of transcription of these three homologues especially in presence of oxygen remains unclear. However, the expressions of these paralogues appeared sequential during growth phases. EF0408_EF0409 was mainly transcribed during exponential phase, EFA0080_EFA0081 during early stationary phase, and EFB0062_EFB0063 after 24 h of stationary phase (Table 3). These observations suggest that the different TA systems may have different roles according to the growth phase of the bacteria.

**Table 3:** Expression patterns of sRNAs under different growth phases and stress conditions.

sRNAs	Stress conditions										
	H_2_O_2_	pH (acid)	BS	Expo	Early Stat	Stat	Expo	Early Stat	Stat	Urine	Serum
				with O_2_	with O_2_	with O_2_					
EF3314_EF3315	127	2263	596	787	1756	64	3478	659	93	191	112
EF0820_EF0822	114	150	135	370	253	47	473	605	51	59	122
EFA0080_EFA0081	102	186	314	857	43619	2364	5034	1566	3886	1756	2178
EF1368_EF1369	761	2817	874	1178	418	118	171	614	168	144	139
EF0408_EF0409	1756	2916	649	20636	1916	283	1597	1909	136	954	257
EF0605_EF0606	722	2056	246	1880	3246	214	261	113	326	802	129
EF1097_EF1098	4535	32765	106115	1835	22301	2438	1518	1492	3977	41662	11483
EF0869_EF0871	556	159	1236	196	7780	13465	374	119683	31710	5974	30293
EF0136_EF0137	59	108	11	70		27	46	11	125	144	101
EFB0062_EFB0063	125	332	194	2817	802	20636	1756	1236	5503	211	179
EF2205_EF2206	10724	21313	11296	9823	25155	10468	202452	11483	405266	29510	221227

*E. faecalis* tmRNA (EF1097_EF1098) and 4.5S RNA (EF2205_EF2206) showed a high intensity of hybridization under all conditions tested but BS and late stationary phase induced the highest level of tmRNA and 4.5S RNA expression, respectively. Furthermore, EF0869_EF0871 was highly expressed in urine and serum medium (Table 3). It has been shown that transcription of some genes encoding fitness and virulence factors are affected when *E. faecalis* is incubated in these biological media [39], [40]. It is then tempting to speculate that these sRNAs could play a crucial role in the cellular response triggered during the infectious process.

Surprisingly, for unexpected reason, signals corresponding to the two sRNAs EF0136_EF0137 and EF0820_EF0822 were very low in these tiling arrays experiments leading to unexploitable data. On the other hand, EF3314_EF3315, EF1368_EF1369 and EF0605_EF0606 sRNAs appeared moderately expressed but were obviously induced by acid stress (Table 3). However, exponential growth phase and early stationary phase in presence of oxygen were the most favorable conditions for EF3314_EF3315 and EF0605_EF0606 expressions, respectively (Table 3). This is in agreement with the induction of Ref25C (corresponding to EF0605_EF0606) in oxidative stress condition reported by Fouquier d'Hérouel *et al.* [23].

Our tiling arrays data using RNA samples obtained from cells incubated under 11 different growth conditions allowed us to identify 76 new IGRs with intensities of hybridization ten fold higher than signals from apparent untranslated regions. *Probe sequences and tiling array data obtained with samples from stressed cells are shown in Table S3. A more detailed analysis of these new candidates is in progress in our laboratory.* In addition, if the threshold was set to five-fold induction, 174 putative sRNAs were detected in our experiments. sRNAs are usually transcribed under specific growth conditions and it is likely that some could be expressed under stressing conditions not yet tested. Moreover, sRNAs may have been missed in our study due to experimental procedure since our chips only covered intergenic regions of the V583 genome and since fractionated RNAs have been used for the hybridizations. It has been generally predicted that genome sizes ranging from 3–4 Mbp may contain 80–300 sRNAs [14]. Taken together it is highly probable that the number of sRNA transcripts detected in *E. faecalis* will greatly increase in the near future.

### Distribution of sRNAs among *E. faecalis* strains

To date, the whole genome sequence of 54 *E. faecalis* strains are available in the NCBI database. We performed standard BLAST analysis to detect the presence of the characterized sRNAs in these different *E. faecalis* strains (Table 4). Seven of them are highly conserved (90 to 100% identical) and present in all *E. faecalis* genomes (EF3314_EF3315, EF0820_EF0821, EF1368_EF1369, EF0408_EF0409, EF1097_EF1098, EF0869_EF0871 and EF2205_EF2206). The other four are not systematically observed because of their location on a mobile genetic element (EF0136_EF0137), in the pathogenicity island (PAI) (EF0605_EF0606) or on plasmids (EFA0080_EF0081 and EFB0062_EFB0063) [41]. sRNAs EF0605_EF0606, EF0136_EF0137, EFA0080_EFA0081 and EFB0062_EFB0063 homologues (at least 80% identical) are present in 9, 15, 35 and 23 strains of the 54 genomes analyzed, respectively (Table 4).

**Table 4:** Distribution of the 11 sRNAs among *E. faecalis* strains.

	sRNAs										
*E. faecalis*	EF3314_	EF0820_	EFA0080_	EF1368_	EF0408_	EF0605_	EF1097_	EF0869_	EF0136_	EFB0062_	EF2205_
strains	EF3315	EF0822	EFA0081	EF1369	EF0409	EF0606	EF1098	EF0871	EF0137	EFB0063	EF2206
OGR1RF	90	100		90	90		90	90			100
ARO1/DG	90	100		90	90		100	90		80–90 P	100
ATCC 29200	100	100	80–90 G	90	90		100	90		80–90 G	100
ATCC 4200	100	100		90	90		100	90			100
CH188	100	100		90	90		100	90	80–90		100
D6	100	100		90	90	100	90	90			100
DAPTP0512	90	100	80–90 G	90	90		90	90		80–90 G	90
DAPTP0516	90	100	80–90 G	90	90		90	90		80–90 G	90
DS5	100	100	100 P	90	90		90	90	80–90	80–90 P	100
E1Sol	100	90		90	90		100	90		80–90 P	100
Fly1	90	90		90	90		90	90			100
HH22	100	100	100 G	90	100		100	100		80–90 G	100
HIP11704	100	90	80–90 G	90	90		90	90		80–90 G	100
JH1	100	100	80–90 P	90	90		90	90	80–90	80–90 P	100
Merz96	90	100	80–90 P	90	90		90	90		80–90 P	90
PC1.1	100	100		90	90		90	90	80–90		100
R712	90	100	80–90 G	90	90		90	90		80–90 G	90
S613	90	100	80–90 G	90	90		90	90		80–90 G	90
T1	100	100		90	90		90	90		90 P	100
T11	100	100		90	100		100	100			100
T2	100	100	80–90 P	90	90	100	90	90	100	80–90 P	100
T3	100	100		90	90		90	90		80–90 P	100
T8	100	100	90 G	90	90		90	90		80–90 G	100
TUSoD Ef11	100	90		80–90	90		90	90			90
TX0012	100	100		90	90		90	90			90
TX0017	100	100	80–90 G	90	90		90	90		80–90 G	100
TX0027	100	100	80–90 G	90	90		90	90	80–90	80–90 G	100
TX0031	100	100		90	90		100	90			100
TX0043	100	100		90	90		100	90			90
TX0102	100	100		90	90		100	90			100
TX0104	100	100	80–90 G	90	90	90	90	90		80–90 G	100
TX0109	100	100		90	90		90	90		80–90 G	90
TX0309A	100	100		90	100	100	100	100	80–90	90 G	100
TX0309B	100	100		90	100	100	100	100	80–90	90 G	100
TX0312	100	100		90	90		100	90			90
TX0411	100	100	80–90 G	90	90		100	90		90 G	90
TX0470	100	100		80–90	90		90	100	80–90		100
TX0630	100	100	80–90 G	90	90		100	90	100	90 G	100
TX0635	100	100	90 G	90	90		100	90	80–90	80–90 G	100
TX0645	100	90		80–90	90		90	90	80–90	80–90 G	100
TX0855	100	90	80–90 G	90	90	100	90	90		90 G	100
TX0860	100	100		90	90	100	90	90		90 G	100
TX1302	100	100		90	90		90	90			100
TX1322	100	100		90	90		100	90		80–90 G	100
TX1341	100	100		90	90	100	90	100	80–90	80–90 G	100
TX1342	100	100		90	90		90	90			100
TX1346	100	90		90	90		90	90			90
TX2134	100	100	80–90 G	90	90		100	90		90 G	100
TX2137	100	100	80–90 G	90	90	100	90	90		80–90 G	100
TX2141	100	90		80–90	90		90	90			90
TX4000	100	100		90	90		90	90			100
TX4244	100	100		90	90		90	90	80–90	80–90 G	100
TX4248	100	100	80–90 G	90	90		90	90	80–90	80–90 G	100
X98	100	100	80–90 P	90	90		100	90		80–90 P	90

100 indicates 100% identity.

90 indicates more than >90% identity.

80–90 indicates between 80 and 90% identity.

White box indicates the absence of homology.

G: on genome.

P: on plasmid.

Homologues of EF0408_EF0409 (more than 90% identity) (member of TA system, see above) were systematically present in all *E. faecalis* genomes. Moreover, additional plasmidic EFA0080_EFA0081 and EFB0062_EFB0063 homologous were also observed in some chromosomes showing that most *E. faecalis* strains have several *par* systems arguing for a selective advantage for the bacterial cell.

Interestingly, EF0605_EF0606 is located in PAI between a gene encoding a Dps family protein (EF_0606) and an operon including a paralogue of *gls24* (EF_0605-EF_0604). Dps is a protein involved in the protection of DNA against oxidative stress and Gls24 corresponds to a general stress protein that is a virulence factor in *E. faecalis* [42], [43], [44]. In *S. pneumoniae*, two sRNAs had demonstrated *cis*-acting effects on the transcription of adjacent genes [45]. From these observations and the fact that EF0605_EF0606 sRNA is induced under aerobic growth conditions, it may be hypothesized that it has a role in the control of expression of these enzymes and hence may be implicated in stress response and virulence of *E. faecalis*.

### Perspectives

In this work we have determined the sequences, locations and expression patterns of 11 sRNAs in *E. faecalis* V583. These results provide a starting point towards understanding of the complex RNA regulatory network governing *E. faecalis* physiology and virulence. Recently, comparative genome-wide analysis of putative or characterized sRNAs of five major Gram-positive pathogens (*L. monocytogenes* EGD-e, *Clostridium difficile* 630, *Staphylococcus aureus* COL, *S. pyrogenes* M1 GAS, and *E. faecalis* V583) was reported [46]. This information will help to understand the molecular mechanisms of the pathogenic process which might be useful for the development of novel microbial diagnosis tools and anti-bacterial drugs such as antisense PNAs (peptide nucleic acids) [46].

## Supporting Information

Table S1 Primers and probes used in this study.
(DOC)

Table S2 List of putative target genes of EF3314_EF3315, EF0820_EF0822, EF1368_EF1369, EF0605_EF0606, EF0869_EF0871, EF0136_EF0137 sRNA candidates.
(XLS)

Table S3 Probe sequences and tiling array data obtained with samples from stressed cells of *E. faecalis*.
(XLS)
