# Supplementary material for: Genome-Wide Identification of Small RNAs in the Opportunistic Pathogen Enterococcus faecalis V583
Source: PLoS One. 2011 Sep 2;6(9):e23948. doi: 10.1371/journal.pone.0023948 (PMC3166299; doi:10.1371/journal.pone.0023948)
Supplement: Table S1 — Primers and probes used in this study. (DOC) [file pone.0023948.s001.doc]

Table S1. Primers and probes used in this study.

| Primer name | Sequence | Note |
| --- | --- | --- |
| EF3314-GSP1 | 5’ AAAAGCAAATCAAGATTCGCTT 3’ | 5’ RACE |
| EF3314-GSP2 | 5’ CAACTGTTTATCATCGTATTTGTAT 3’ | 5’ RACE and Northern probe |
| EF3314-GSP3 | 5’ ACTCTGATAAGAATGATTTCGT 3’ | 5’ RACE |
| EF3314-GSP4 | 5’ GAAACACAGTATATTTTATT 3’ | 3’ RACE |
| EF3314-GSP5 | 5’ ACGAAATCATTCTTATCAGAGT 3’ | 3’ RACE |
| EF0820-GSP1 | 5’ GGCAACACTCATGGGAATG 3’ | 5’ RACE |
| EF0820-GSP2 | 5’ GTTCATTGTTATCGTTGTTCTC 3’ | 5’ RACE |
| EF0820-GSP3 | 5’ TCCCAGGTCATTACGCTGT 3’ | 5’ RACE and Northern probe |
| EF0820-GSP4 | 5’ ACAGCGTAATGACCTGGGA 3’ | 3’ RACE |
| EF0820-GSP5 | 5’ GAGAACAACGATAACAATGAAC 3’ | 3’ RACE |
| EFA0080-GSP1 | 5’ AGCAATCCTATTCGCCGTA 3’ | 5’ RACE |
| EFA0080-GSP2 | 5’ GGATTGCTTTTTGTGTTATCTGT 3’ | 5’ RACE and Northern probe |
| EFA0080-GSP3 | 5’ ACGATTTAATGTCGTTTCGC 3’ | 5’ RACE |
| EFA0080-GSP4 | 5’ GCGAAACGACATTAAATCGT 3’ | 3’ RACE |
| EFA0080-GSP5 | 5’ ACAGATAACACAAAAAGCAATCC 3’ | 3’ RACE |
| EF1368-GSP1 | 5’ ACAAAAAAAACCACCACGACC 3’ | 5’ RACE |
| EF1368-GSP2 | 5’ GTCGTCTGTGGTGGAAATGA 3’ | 5’ RACE |
| EF1368-GSP3 | 5’ AGGTTGTTATTTACTCTTGGTA 3’ | 5’ RACE and Northern probe |
| EF1368-GSP4 | 5’ ATTGGTTTTTTCATACTTACTCC 3’ | 3’ RACE |
| EF1368-GSP5 | 5’ TCATTTCCACCACAGACGAC 3’ | 3’ RACE |
| EF0408-GSP1 | 5’ GGTGTCTTTTTAGTGCATCC 3’ | 5’ RACE |
| EF0408-GSP2 | 5’ ATGCACGACTTTGTCACAAG 3’ | 5’ RACE and Northern probe |
| EF0408-GSP3 | 5’ TTACTTTAAATTTATCATAGGGCCT 3’ | 5’ RACE |
| EF0408-GSP4 | 5’ CTTGTGACAAAGTCGTGCAT 3’ | 3’ RACE |
| EF0605-GSP1 | 5’ AGTGACCGATTTCAGTCGTT 3’ | 5’ RACE |
| EF0605-GSP2 | 5’ AAATATAAGTACAGATAGATTTTATCA 3’ | 5’ RACE |
| EF0605-GSP3 | 5’ AACTAATTACATAAAAAAGGATATGA 3’ | 5’ RACE |
| EF0605-GSP4 | 5’ TCTCTAGGGGTGACTCAGAG 3’ | 3’ RACE |
| EF0605-GSP5 | 5’ CAGGGGAAGGAGTTACCCCT 3’ | 3’ RACE |
| EF0605 | 5’ CTCTGAGTCACCCCTAGAGA 3’ | Northern probe |
| EF1097-GSP1 | 5’ AGACGGCGGGAGTCGAA 3’ | 5’ RACE |
| EF1097-GSP2 | 5’ GCGTCCAAACATATTGCCAC 3’ | 5’ RACE and Northern probe |
| EF1097-GSP3 | 5’ TTAAATCTCTACGTTCATAGACA 3’ | 5’ RACE |
| EF1097-GSP4 | 5’ TCGAAGTGGGATACGCTAAA 3’ | 3’ RACE |
| EF1097-GSP5 | 5’ TAGAGGAGCTTACCAGACTA 3’ | 3’ RACE |
| EF0869-GSP1 | 5’ ACGCTTGGATGGTGGATAACT 3’ | 5’ RACE |
| EF0869-GSP2 | 5’ TGCGTTAGGTGATAACAGAT 3’ | 5’ RACE |
| EF0869-GSP3 | 5’ AGGGTAAATGCTTTATAGAAACTCCA 3’ | 5’ RACE |
| EF0869-GSP4 | 5’ CTATTCTTTAAGTGTAAGGAGGA 3’ | 3’ RACE |
| EF0869-GSP5 | 5’ CATGCAAATCAATTGGTTGAAG 3’ | 3’ RACE |
| EF0869 | 5’ CTTCAACCAATTGATTTGCATG 3’ | Northern probe |
| EF0136-GSP1 | 5’ GCAATGGGTTAGTGTTTATATATT 3’ | 5’ RACE |
| EF0136-GSP2 | 5’ GTTTAGCTTGATTTGCTGTTC 3’ | 5’ RACE and Northern probe |
| EF0136-GSP3 | 5’ TATAATGGGAGTAGAATAGGC 3’ | 5’ RACE |
| EF0136-GSP4 | 5’ TGCTTCGCTTCTAGCATAGGA 3’ | 3’ RACE |
| EF0136-GSP5 | 5’ ACCAAAAATGTAGATTACTGGAACA 3’ | 3’ RACE |
| EFB0062-GSP1 | 5’ AGGTTTGGGCTATTGTGC 3’ | 5’ RACE |
| EFB0062-GSP2 | 5’ TGTTCATATCGAACTTATGTCT 3’ | 5’ RACE and Northern probe |
| EFB0062-GSP3 | 5’ TCCGCATTATTAGTATAACACATT 3’ | 5’ RACE |
| EFB0062-GSP4 | 5’ AATGTGTTATACTAATAATGCGGA 3’ | 3’ RACE |
| EFB0062-GSP5 | 5’ AGACATAAGTTCGATATGAACA 3’ | 3’ RACE |
| EF2205-GSP1 | 5’ GATACGTTCCTTATTCAATCAGACA 3’ | 5’ RACE |
| EF2205-GSP2 | 5’ CATGGCGGCACTTGCTTAGT 3’ | 5’ RACE |
| EF2205-GSP3 | 5’ CTGCTTCCTTCCGGATCTG 3’ | 5’ RACE and Northern probe |
| EF2205-GSP4 | 5’ TGCCGAAAGGCTAGGACAAT 3’ | 5’ RACE |
| EF2205-GSP5 | 5’ GGCGGGCTAGTGAATTGTGT 3’ | 3’ RACE |
| EF3314U5' | 5’ TTCGGATCCGTCAAATCAATATCAACACC 3’  *Bam*HI | Cloning in pMAD |
| EF3314U3' | 5’ CAGGTCGACCGTTATTTAATTATCGATTTATCAAA 3’  *Sal*I | Cloning in pMAD |
| EF3314D5' | 5’ TCTGTCGACTTTCCATAGGCATATAAGGTCA 3’  *Sal*I | Cloning in pMAD |
| EF3314D3' | 5’ GATGAATTCGATGCAGGCGCCCATCA 3’  *Eco*RI | Cloning in pMAD |
| *madF* | 5’ TCTAGCTAATGTTACGTTACAC 3’ | Cloning and plasmid verification |
| *madR* | 5’ TCATAATGGGGAAGGCCATC 3’ | Cloning and plasmid verification |
